# New Scoring System for Prediction of Surgical Difficulty During Laparoscopic Cholecystectomy After Percutaneous Transhepatic Gallbladder Drainage

**DOI:** 10.1002/ags3.12522

**Published:** 2021-10-27

**Authors:** Michinori Matsumoto, Kyohei Abe, Yasuro Futagawa, Kenei Furukawa, Koichiro Haruki, Shinji Onda, Takanori Kurogochi, Nana Takeuchi, Tomoyoshi Okamoto, Toru Ikegami

**Affiliations:** ^1^ Department of Surgery The Jikei University Daisan Hospital Komae Japan; ^2^ Department of Surgery The Jikei University School of Medicine Minato‐ku Japan

**Keywords:** bile duct injury, laparoscopic cholecystectomy, percutaneous transhepatic gallbladder drainage, receiver operating characteristic, scoring system

## Abstract

**Background:**

The surgical difficulty of laparoscopic cholecystectomy (LC) after percutaneous transhepatic gallbladder drainage (PTGBD) remains unknown. This study aimed to establish a scoring system (SS) to predict the necessity of a bailout procedure during LC after PTGBD and to evaluate the relationship between SS and perioperative complications.

**Methods:**

We retrospectively studied 70 patients who underwent LC after PTGBD. Preoperative factors potentially predictive of the need for the bailout procedure were analyzed. The SS included significantly predictive factors, with their cutoff values determined by receiver operating characteristic curves. Patients were assigned a score of 1 when exhibiting only one of these abnormalities. We compared the perioperative factors between three groups with scores of 0, 1, or 2. The SS was applied to another series of 65 patients for validation. We compared the score‐2 patient perioperative factors between LC with the bailout procedure and open cholecystectomy from the beginning (OC).

**Results:**

Independent predictors were time until PTGBD after symptom onset and the maximal wall gallbladder thickness (cutoff values: 3 days and 10 mm, respectively). The high‐score group was significantly associated with bile duct injury (BDI). The sensitivity and specificity of our SS were 75.0% and 98.1% in validation, respectively. The score‐2 OC and laparoscopic subtotal cholecystectomy (LSC) groups had no BDI.

**Conclusions:**

The SS using time until PTGBD after symptom onset and gallbladder wall thickness for predicting the need for the bailout procedure correctly predicted the need. The scores might be associated with the risk of BDI, and LSC or OC might be a better choice for score‐2 patients.

## Graphical abstract

We propose a new scoring system to predict the necessity of a bailout procedure during LC after PTGBD, using significant predictors of time until PTGBD after symptom onset and the maximal wall thickness of GB. Our scoring system for the necessity of the bailout procedure seems to provide a precise prediction and might be associated with the risk of BDI. It might be better to select flexibly laparoscopic subtotal cholecystectomy or open cholecystectomy from the beginning for patients with a score of 2 after PTGBD.

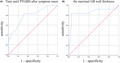

## 1 INTRODUCTION

Laparoscopic cholecystectomy (LC) has become a standard procedure for benign diseases of the gallbladder (GB) worldwide.^1^ Severe inflammation of GB and its surroundings increases both the difficulty of complete LC and the frequency of postoperative complications.^2^ Bile duct injury (BDI) is known to occur in a certain proportion of cases, and the prognoses of patients who suffer vasculo‐biliary injury (VBI) in particular are poor.^3^ Therefore, it is very important to take prudent steps to prevent complications.^4^ The Tokyo Guidelines 2018 (TG18) propose management bundles for acute cholecystitis (AC) and cholangitis. When LC for AC is difficult, not only open conversion but also laparoscopic subtotal cholecystectomy (LSC) with the fenestrating or reconstituting and fundus first technique, called the bailout procedure, can be chosen to prevent BDI according to the intraoperative findings.^4^

On the other hand, early surgery for AC cannot be performed for all surgically high‐risk patients. Percutaneous transhepatic GB drainage (PTGBD) should be considered the first alternative to cholecystectomy in surgically high‐risk patients with AC because several studies have described PTGBD as less invasive and having a lower risk of adverse events than cholecystectomy.^5^ However, the degree of surgical difficulty during LC after PTGBD is unknown, and no report has provided scientific evidence of the conditions supporting use of the bailout procedure during LC after PTGBD.

This study aimed to establish a scoring system (SS) to predict the necessity of the bailout procedure during LC after PTGBD and to evaluate the relationship between the SS and perioperative complication.

## 2 PATIENTS AND METHODS

**FIGURE 1:**
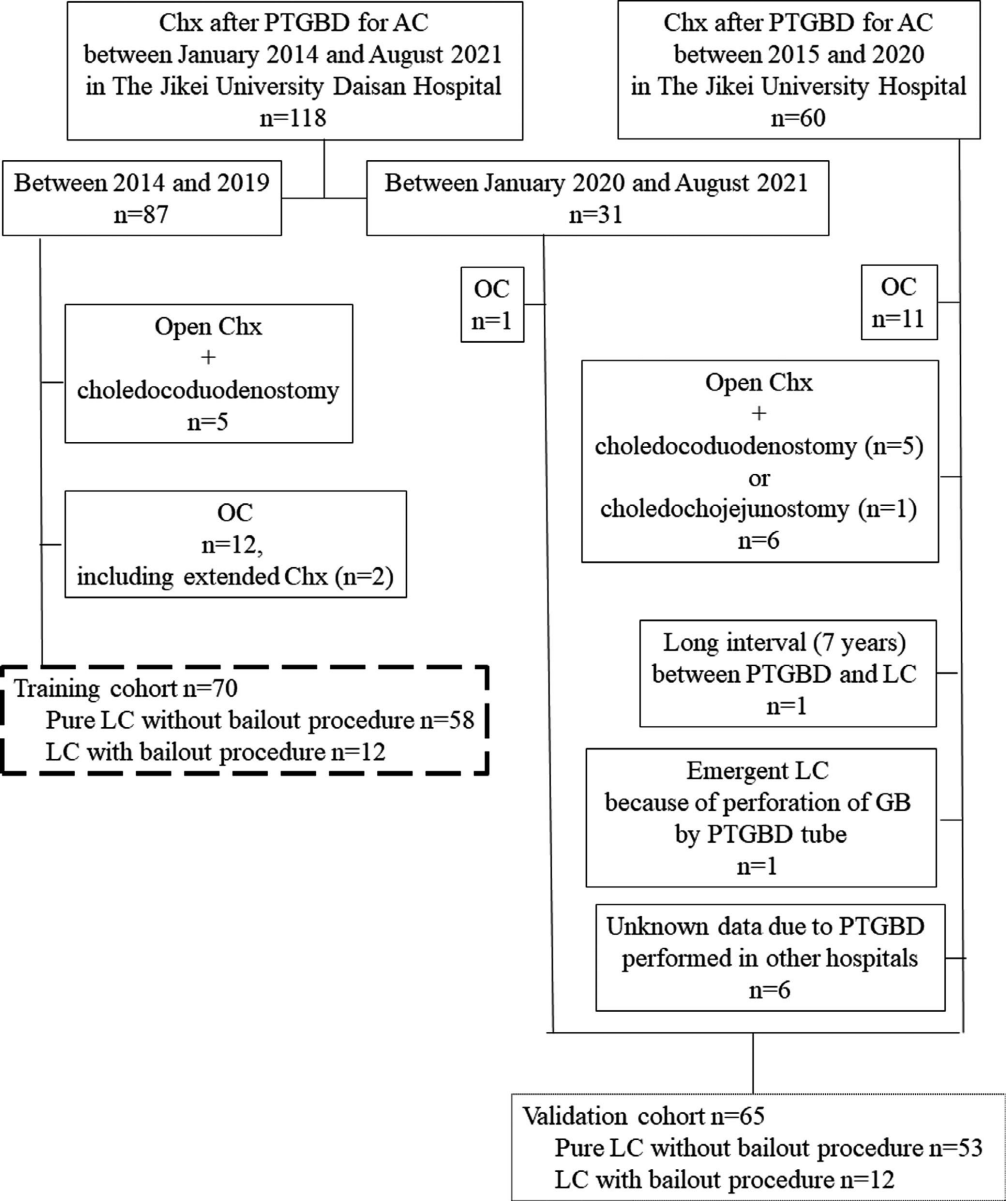
Flow diagram describing the patient selection process. AC, acute cholecystitis; PTGBD, percutaneous transhepatic gallbladder drainage; LC, laparoscopic cholecystectomy; Chx, cholecystectomy; OC, open cholecystectomy from the beginning

The medical records of a series of 178 consecutive patients who had undergone LC or open cholecystectomy after PTGBD for AC between January 2014 and August 2021 in The Jikei University Daisan Hospital and The Jikei University Hospital were retrospectively reviewed. The 178 patients were divided into two groups, one for model development (training cohort) and the other for validation testing (validation cohort). We defined open conversion from LC or LSC with the fenestrating or reconstituting technique as the bailout procedure. In the first 87 patients for the training cohort between 2014 and 2019 in The Jikei University Daisan Hospital, 12 patients who had undergone open cholecystectomy from the beginning (OC) and five patients who had undergone OC with choledochoduodenostomy for cholodocholithiasis were excluded. The remaining 70 patients were divided into 58 patients who had undergone pure LC without the bailout procedure and 12 patients who had undergone LC with the bailout procedure. Independent factors predictive of the necessity of the bailout procedure were examined for the training cohort of the 70 patients. In the validation cohort operated in The Jikei University Daisan Hospital between January 2020 and August 2021 and in The Jikei University Hospital between 2015 and 2020, 12 patients who had undergone OC, five patients who had undergone open cholecystectomy with choledochoduodenostomy for choledocholithiasis, one patient who had undergone open cholecystectomy with choledochojejunostomy for choledocholithiasis, one patient who had undergone LC 7 years after PTGBD, and six patients with unknown data due to PTGBD in other hospitals were excluded. The remaining 65 patients were divided into 53 patients who had undergone pure LC without the bailout procedure and 12 patients who had undergone LC with the bailout procedure. The 65 patients were examined to assess the accuracy of our SS (Figure 1).

Most of PTGBD procedures were performed by physicians in our hospital. The main indication of PTGBD in our hospital was Grade II (moderate) or III (severe) AC according to TG18^5^ when the patients could not withstand surgery and were AC refractory to antibiotics. Measurement of the maximum GB wall thickness was performed on the axial or coronal plane of noncontrast or contrast‐enhanced computed tomography just before PTGBD because most of the images of abdominal ultrasonography just before or during PTGBD had not been stored.

The following 14 preoperative factors of these patients were analyzed to predict the necessity of the bailout procedure during LC in univariate and multivariate analysis: age, gender, body mass index, procedure (single‐port or multi‐port LC), American Society of Anesthesiologists physical status classification, age‐adjusted Charlson comorbidity index (CCI),^6^, ^7^ anticoagulant therapy, past history of upper abdominal surgery, time until PTGBD after symptom onset, time until surgery after PTGBD, maximal GB wall thickness, maximal diameter of impacted stone in the GB, maximal white blood cell (WBC) count in the peripheral blood, and maximal serum value of C‐reactive protein. The cutoff score of CCI was determined based on a past report.^8^

The SS was designed by using the significant predictive factors, the cutoff values of which were determined by a receiver operating characteristic (ROC) curve. Patients were assigned to a score of 2 if they had two significant factors predictive of both abnormalities for the respective cutoff values, a score of 1 if they only had one factor predictive of these abnormalities, and a score of 0 if neither abnormality was present. We then compared the 18 perioperative factors, which are the previous 14 preoperative factors and another four perioperative factors (open conversion, subtotal cholecystectomy, BDI, and postoperative complication) among three groups with scores of 0, 1, and 2, respectively. The SS was applied to another series of 65 patients for validation.

Finally, we compared the 21 perioperative factors, which are the previously described 18 perioperative factors and another three operative factors (LSC with fenestrating or reconstituting, operation time, and estimated blood loss during operation) between patients with a score of 2 between 2014 and August 2021 in The Jikei University Daisan Hospital and between 2015 and 2020 in The Jikei University Hospital who underwent LC with the bailout procedure and those who underwent OC.

This retrospective study was approved by the Ethics Committee of The Jikei University School of Medicine (Tokyo, Japan); 27‐177 (8062).

### 2.1 Statistical methods

Continuous data were expressed as the median and range, and compared by the Mann–Whitney *U*‐test between two groups and by the Kruskal–Wallis test among three groups. Categorical data were compared by the chi‐square test. Univariate factors predictive of the need for the bailout procedure (*P* < .05) were entered into a logistic regression model to identify the independent conversion predictors. Multivariate analysis was performed by a stepwise backward procedure. The accuracy of these independent predictors for the bailout procedure were evaluated by calculating the area under ROC curve (AUROC). The ROC curve was a plot of sensitivity vs 1 – specificity for all possible cutoff values. Cutoff values of these independent predictors were determined by an ROC curve. *P* < .05 was considered to be indicative of statistical significance in all analyses.

## 3 RESULTS

### 3.1 Characteristics of the clinical patients

The characteristics of the 70 patients are summarized in Table 1. For all cases, there were 51 men and 19 women. The median age was 73 years (range, 28–89 years). All patients who underwent the bailout procedure had open conversion form LC. The conversion rate was 17% (=12/70). The reasons for conversion were as follows: difficulty in dissection at Calot's triangle in five patients, difficulty in dissection for adhesion to the duodenum and/or transverse colon in six patients, and BDI in one patient. Subtotal cholecystectomy was performed for five patients after open conversion; fenestrating in three patients, reconstituting in one patient, and both in one patient. There were three patients with BDI in this training cohort. Of the three cases, one had BDI and another had VBI with injury to the right hepatic artery after open conversion. The other patient had BDI during LC.

**TABLE 1:** Patient characteristics in the training cohort

	Factor 〔Median (minimum to maximum) or Ratio〕	All cases	Pure LC without bailout procedure	LC with bailout procedure	*P*‐value
		(n = 70)	(n = 58)	(n = 12)	
Preoperative factor	Age (years old)	73 (28–89)	73.5 (28–89)	68 (43–83)	.809
	Gender (male : female)	51:19	41:17	10:2	.370
	BMI	23.9 (16. 4–32.8)	24.0 (16.6–32.8)	23.6 (16.4–28.4)	.839
	SILS (yes : no)	5:65	4:54	1:11	.860
	ASA‐PS (1:2:3)	13:51:6	12:42:4	1:9:2	.380
	CCI (≤5:≤6)	50:20	41:17	9:3	.764
	HT (yes:no)	38:32	30:28	8:4	.344
	DM (yes:no)	19:51	16:42	3:9	.854
	Past history of upper abdominal surgery (yes:no)	3:67	3:55	0:12	.421
	Anticoagulative therapy (yes:no)	17:53	15:43	2:10	.499
	Time until PTGBD after symptom onset (days)	2 (0–16)	2 (0–8)	4 (1–16)	.001
	Time until surgery after PTGBD (days)	64.5 (3–281)	64.5 (3–281)	61 (13–102)	.969
	GB wall thickness (mm)	5 (2–21)	5 (2–15)	11.5 (2–21)	.001
	Maximal diameter of impacted stone (mm)	4 (0–40)	4 (0–40)	7 (3–29)	.093
	Maximal WBC count in the peripheral blood (/μL)	13,450 (6800–31,800)	13,000 (6800–27,200)	17,350 (10 800–31,800)	.031
	Maximal serum value of CRP (mg/dL)	19.8 (0.5–40.4)	19.8 (0.5–39.2)	23.3 (14.5–40.4)	.052
Intra‐ or postoperative factor	Open conversion (yes:no)	12:58	0:58	12:0	<.0001
	LSC with fenestrating or reconstituting (yes:no)	0:70	0:58	0:12	NA
	Subtotal cholecystectomy with fenestrating or reconstituting (yes:no)	5:65	0:58	5:7	<.0001
	BDI during operation (yes:no)	3:0	0:58	3:9	<.0001
	Grade of Clavien–Dindo classification ≤3 (yes:no)	2:68	1:57	1:11	.211

Abbreviations: ASA‐PS, the American Society of Anesthesiologists physical status classification; BDI, bile duct injury; BMI, body mass index; CCI, Age‐adjusted Charlson comorbidity index; CRP, C‐reactive protein; DM, diabetes mellitus; GB, gallbladder; HT, hypertension; LC, laparoscopic cholecystectomy; LSC, laparoscopic subtotal cholecystectomy; NA, not available; PTGBD, percutaneous transhepatic gallbladder drainage; SILS, single‐port laparoscopic surgery; WBC, white blood cell.

### 3.2 Univariate and multivariate predictors of the conversion

Significant univariate predictors of the necessity of the bailout procedure during LC after PTGBD were time until PTGBD after symptom onset (*P* = .001), the maximal GB wall thickness (*P* = .001), and the maximal WBC count in the peripheral blood (*P* = .031) (Table 1). Time until PTGBD after symptom onset and the maximal GB wall thickness were independent clinical predictors for the necessity of the bailout procedure during LC after multivariate analysis (odds ratio [OR] = 1.416, 95% confidence interval [CI]: 1.005–1.994, *P* = .046 and OR = 1.423, 95% CI: 1.135–1.784, *P* = .002, respectively) (Table 2).

**TABLE 2:** Logistic regression for independently predicted factors of necessity of the bailout procedure during LC after PTGBD

Predictor	OR	Regression coefficient	95% CI	SE	*P*‐value
Time until PTGBD after symptom onset (days)	1.416	0.348	1.005–1.994	0.175	.046
GB wall thickness (mm)	1.423	0.353	1.135–1.784	0.115	.002

Abbreviations: CI, confidence interval; GB, gallbladder; LC, laparoscopic cholecystectomy; OR, odds ratio; PTGBD, percutaneous transhepatic gallbladder drainage; SE, standard error.

### 3.3 Determination of cutoff values for the independent predictors

**FIGURE 2:**
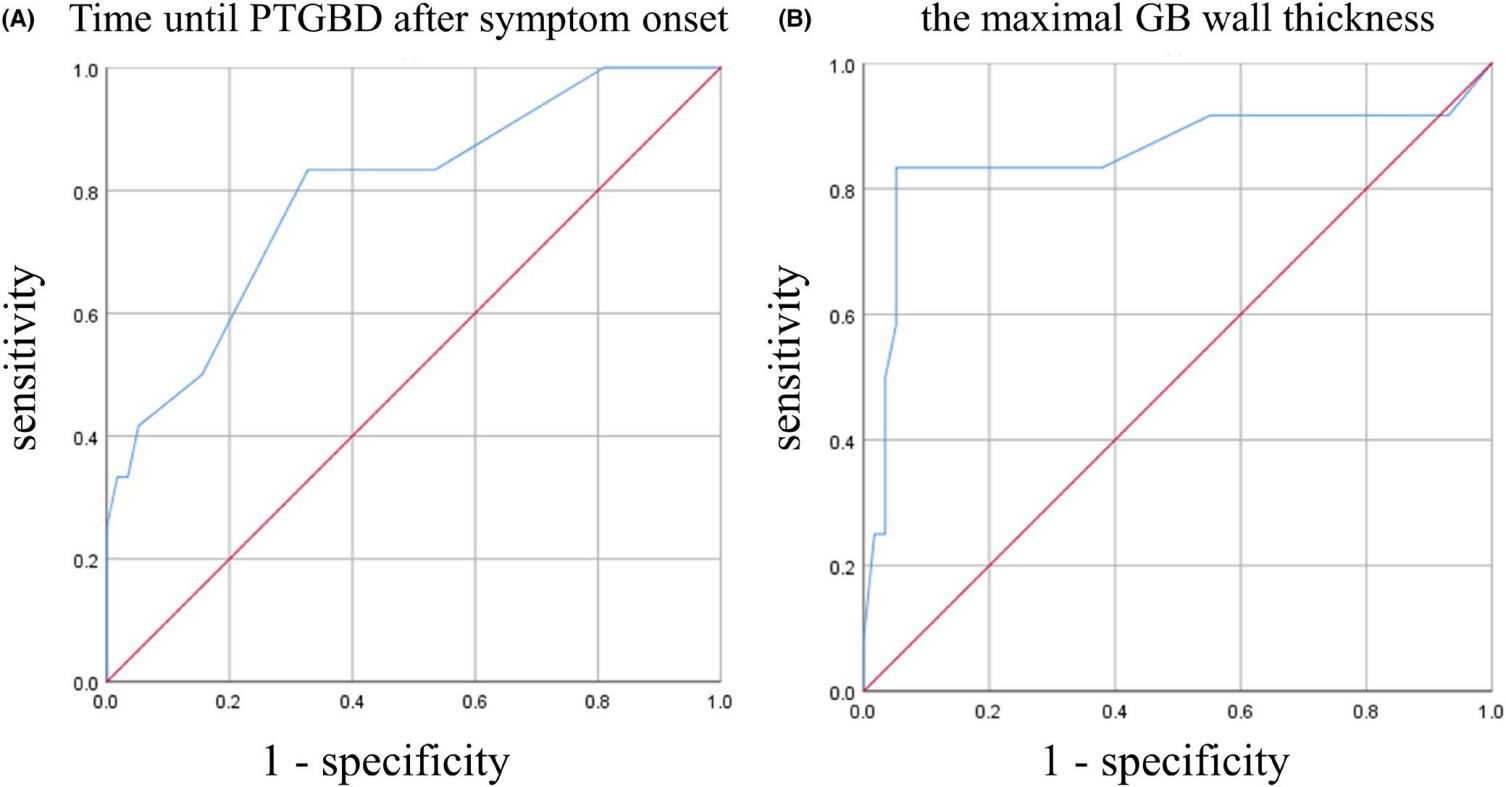
Receiver operating characteristic (ROC) curves of the time until PTGBD after symptom onset (A) and the maximal GB wall thickness (B) for predicting the bailout procedure during laparoscopic cholecystectomy. The blue line shows an ROC curve. The red line shows the reference line. (A) Cutoff value, 3 d; sensitivity, 0.833; 1 ‐ specificity, 0.328; AUROC, 0.795; 95% CI, 0.650–0.939; SE, 0.074; *P* = .001. (B) Cutoff value, 10 mm; sensitivity, 0.833; 1 ‐ specificity, 0.053; AUROC, 0.854; 95% CI, 0.694–1.000; SE, 0.082; *P* < .0001. PTGBD, percutaneous transhepatic gallbladder drainage; GB, gallbladder; AUROC, area under receiver operating characteristic curve; CI, confidence index; SE, standard error

The results of the ROC curve of time until PTGBD after symptom onset and the maximal GB wall thickness are shown in Figure 2. The maximal sensitivity and specificity were obtained when the time until PTGBD after symptom onset and the maximal GB wall thickness were 3 d and 10 mm, respectively (AUROC = 0.795, 95% CI: 0.650–0.939, *P* = .001 and AUROC = 0.854, 95% CI: 0.694–1.000. *P* < .0001, respectively) (Figure 2).

### 3.4 Univariate analysis of the relationship between patient characteristics and the SS

Two variables were used in the design of the SS. In brief, patients were assigned a score of 2 if they had both a longer time until PTGBD after symptom onset (≥3 days) and a thicker GB wall (≥10 mm), a score of 1 when exhibiting only one of these abnormalities, and a score of 0 if neither abnormality was present (Table 3). This SS design was significantly associated with the open conversion during LC (*P* < .0001) and subtotal cholecystectomy with the fenestrating or reconstituting technique (*P* < .0001) (Table 4). On the other hand, patients with high scores were significantly associated with BDI (*P* < .0001) (Table 4).

**TABLE 3:** The scoring system for the prediction of the necessity of the bailout procedure during LC

Factors	Point 0	Point 1
Time until PTGBD after symptom onset	<3 days	3 days≤
GB wall thickness	<10 mm	10 mm ≤

Abbreviations: GB, gallbladder; LC, laparoscopic cholecystectomy; PTGBD, percutaneous transhepatic gallbladder drainage.

**TABLE 4:** Patient characteristics in relationship to a scoring system

	Factor 〔Median (minimum to maximum) or Ratio〕	Score 0	Score 1	Score 2	*P*‐value
		(n = 38)	(n = 21)	(n = 11)	
Preoperative factor	Age (years old)	73 (37–89)	75 (37–83)	65 (28–82)	.264
	Gender (male:female)	26:12	17:4	8:3	.584
	BMI	23.0 (16.6–31.5)	24.8 (19.5–32.8)	24.1 (16.4–28)	.229
	SILS (yes:no)	1:37	2:19	2:9	.186
	ASA‐PS (1:2:3)	7:28:3	4:15:2	2:8:1	1.000
	CCI (≤5:≤6)	27:11	14:7	9:2	.664
	HT (yes:no)	18:20	13:8	7:4	.447
	DM (yes:no)	10:28	7:14	2:9	.648
	Past history of upper abdominal surgery (yes:no)	2:36	1:20	0:11	.744
	Anticoagulative therapy (yes:no)	9:29	6:15	2:9	.802
	Time until PTGBD after symptom onset (days)	1 (0–2)	3 (1‐8)	4 (3–16)	<.0001
	Time until surgery after PTGBD (days)	69 (3–281)	60 (5–109)	55 (4–102)	.36
	GB wall thickness (mm)	4 (2–9)	5 (2–15)	12 (10–21)	<.0001
	Maximal diameter of impacted stone (mm)	4 (0–60)	4 (0–16)	5 (0–29)	.906
	Maximal WBC count in the peripheral blood (/μL)	13,050 (6800–27,200)	13,600 (7000–22,500)	13,700 (9800–31,800)	.578
	Maximal serum value of CRP (mg/dL)	18.2 (2.7–33.9)	24.2 (6.5–40.4)	19.8 (0.5–36.0)	.322
Intra‐ or postoperative factor	Open conversion (yes:no)	1:37	2:19	9:2	<.0001
	Subtotal cholecystectomy fenestrating or reconstituting (yes:no)	0:38	1:20	4:7	<.0001
	BDI during operation (yes:no)	0:38	0:21	3:8	<.0001
	Grade of Clavien–Dindo classification ≤3 (yes:no)	0:38	1:20	1:10	.231

Abbreviations: ASA‐PS, the American Society of Anesthesiologists physical status classification; BDI, bile duct injury; BMI, body mass index; CCI, Age‐adjusted Charlson comorbidity index; CRP, C‐reactive protein; DM, diabetes mellitus; GB, gallbladder; HT, hypertension; LSC, laparoscopic subtotal cholecystectomy; PTGBD, percutaneous transhepatic gallbladder drainage; SILS, single‐port laparoscopic surgery; WBC, white blood cell.

### 3.5 Validation of the SS for prediction of the bailout procedure

The frequency distribution of the necessity of the bailout procedure during LC according to the time until PTGBD after symptom onset and the maximal GB wall thickness was examined in another series of 65 patients. Table 5 shows the patient characteristics of the 65 patients; of these, 27, 28, and 10 patients had scores of 0, 1, and 2, respectively. Three patients with a score of <2 underwent the bailout procedure during LC. The reasons for conversion were as follows: difficulty in dissection at Calot's triangle in four patients, difficulty in dissection for adhesion to the duodenum and/or transverse colon in two patients, and BDI in one patient. One of the patients who underwent LSC was diagnosed with GB cancer by histopathological examination after surgery. This patient underwent the additional liver bed resection and biliary resection and reconstruction. In univariate analysis, the GB wall in the LC with the bailout procedure group was significantly thicker than the GB wall in the pure LC without the bailout procedure group (*P* = .003). The percentage of patients who required the bailout procedure during LC was 75.0% in patients with a score of 2 (*P* < .0001). The SS was designed using these two variables. By comparing patients with a score of 2 with those with a score of 1 and less, the sensitivity and specificity of our SS for prediction of the bailout procedure were 75.0% (=9/12) and 98.1% (=52/53), respectively (Table 5).

**TABLE 5:** Patient characteristics in the validation cohort

	Factor [Median (minimum to maximum) or Ratio]	All cases	Pure LC without bailout procedure	LC with bailout procedure	*P*‐value
		(n = 65)	(n = 53)	(n = 12)	
Preoperative factor	Age (years old)	68 (33–91)	68 (33–91)	67 (46–90)	.852
	Gender (male:female)	48:17	38:15	10:2	.408
	BMI	23.0 (7.9–35.0)	23.1 (7.9–35.0)	22.2 (18.4–30.0)	.618
	SILS (yes:no)	0:65	0:53	0:12	NA
	ASA (1:2:3)	12:47:6	10:38:5	2:9:1	.974
	CCI (≤5:≤6)	56:9	45:8	11:1	.540
	HT (yes:no)	28:37	23:30	5:7	.913
	DM (yes:no)	21:44	18:35	3:9	.549
	Past history of upper abdominal surgery (yes:no)	2:63	1:52	1:11	.243
	Anticoagulative therapy (yes:no)	12:53	11:42	1:11	.317
	Time until PTGBD after symptom onset (days)	2 (0–13)	2 (0–11)	3.5 (1–13)	.047
	Time until surgery after PTGBD (days)	57 (1–387)	57 (4–387)	55 (1–345)	.819
	GB wall thickness (mm)	8 (3–16)	7 (3–14)	10.5 (4–16)	.003
	Maximal diameter of impacted stone (mm)	5 (0–48)	5 (0–32)	7.5 (2–48)	.064
	Maximal WBC count in the peripheral blood (/μL)	14 800 (6300–39,300)	14 800 (6300–25,300)	14 550 (8700–39,300)	.833
	Maximal serum value of CRP (mg/dL)	21.86 (1.05–39.24)	23.19 (1.05–39.24)	19.03 (13.25–36.04)	.784
	Score for prediction of the bailout procedure during LC (0:1:2)	27:28:10	24:28:1	3:0:9	<.0001
	Score for prediction of the bailout procedure during LC (0 or 1:2)	55:10	52:1	3:9	<.0001
Intra‐ or postoperative factor	Open conversion (yes:no)	7:58	0:53	7:5	<.0001
	LSC with fenestrating or reconstituting (yes:no)	4:61	0:53	5:7	<.0001
	Subtotal cholecystectomy with fenestrating or reconstituting (yes:no)	6:59	0:53	7:5	<.0001
	BDI during operation (yes:no)	1:64	0:53	1:11	.034
	Grade of Clavien–Dindo classification ≤3 (yes:no)	5:65	4:49	1:11	.236

Abbreviations:ASA‐PS, the American Society of Anesthesiologists physical status classification; BDI, bile duct injury; BMI, body mass index; CCI, Age‐adjusted Charlson comorbidity index; CRP, C‐reactive protein; DM, diabetes mellitus; GB, gallbladder; HT, hypertension; LC, laparoscopic cholecystectomy; LSC, laparoscopic subtotal cholecystectomy; NA, not available; PTGBD, percutaneous transhepatic gallbladder drainage; SILS, single‐port laparoscopic surgery; WBC, white blood cell.

### 3.6 Univariate analysis of patient characteristics between patients with a score of 2 from 2014 to August 2021 who underwent LC with the bailout procedure and who underwent OC

Thirteen patients had OC after PTGBD between 2014 and August 2021 in The Jikei University Daisan Hospital and 11 patients had OC after PTGBD between 2015 and 2020 in The Jikei University Hospital. Among the total 24 patients who had OC, nine patients had a score of 2. Table 6 showed that although there was no significant difference between the patients who underwent LC with the bailout procedure and those who underwent OC with a score of 2, none of the patients with a score of 2 who underwent the OC had BDI (*P* = .125). In addition, among patients with a score of 2 who underwent LC with the bailout procedure, only patients who underwent open conversion had BDI while patients who underwent LSC had no BDI.

**TABLE 6:** Patient characteristics with a score of 2

	Factor 〔Median (minimum to maximum) or Ratio〕	OC	LC with bailout procedure		*P*‐value
		(n = 9)	(n = 18)		
			LSC	Open conversion	
			(n = 4)	(n = 14)	
Preoperative factor	Age (years old)	73 (39–91)	66.5 (43–90)		0.705
	Gender (male:female)	7:2	14:4		1.000
	BMI	21.0 (17.1–41.0)	23.8 (16.4–30.0)		0.495
	SILS (yes:no)	0:9	1:17		0.471
	ASA–PS (1:2:3)	1:7:1	1:16:1		0.746
	CCI ( ≤ 5:6 ≤)	7:2	15:3		0.726
	HT (yes:no)	4:5	12:6		0.268
	DM (yes:no)	2:7	4:14		1.000
	Past history of upper abdominal surgery (yes:no)	2:7	0:18		0.038
	Anticoagulative therapy (yes:no)	3:6	3:15		0.326
	Time until PTGBD after symptom onset (days)	4 (3–17)	5 (3–16)		0.860
	Time until surgery after PTGBD (days)	29 (5–232)	53.5 (5–102)		0.348
	Gallbladder wall thickness (mm)	13 (10–25)	12 (10–21)		0.312
	Maximal diameter of impacted stone (mm)	8 (0–33)	6.5 (2–29)		0.781
	Maximal WBC count in the peripheral blood (/μL)	16 500 (12 200–20,400)	14 700 (8700–39,300)		0.860
	Maximal serum value of CRP (mg/dL)	25.03 (13.30–31.08)	20.65 (13.25–36.04)		0.348
Intra‐ or postoperative factor	Op time (min)	131 (82–514)	164.5 (80–420)		0.176
	Estimated blood loss during operation (g)	105 (5–510)	100 (5‐1,350)		0.668
	Open conversion (yes:no)	0:9	14:4		<0.0001
	LSC with fenestrating or reconstituting (yes:no)	0:9	4:14		0.125
	Subtotal cholecystectomy with fenestrating or reconstituting (yes:no)	3:6	10:8		0.276
	BDI during operation between two groups (yes:no)	0:9	4:14		0.125
	BDI during operation among three groups (yes:no)	0:9	0:4	4:10	0.113
	Grade of Clavien–Dindo classification ≤3 between two groups (yes:no)	0:9	2:16		0.299
	Grade of Clavien–Dindo classification ≤3 among three groups (yes:no)	0:9	0:4	2:12	0.367

Abbreviations: ASA‐PS, the American Society of Anesthesiologists physical status classification; BDI, bile duct injury; BMI, body mass index; CCI, Age‐adjusted Charlson comorbidity index; CRP, C‐reactive protein; DM, diabetes mellitus; GB, gallbladder; HT, hypertension; LC, laparoscopic cholecystectomy; LSC, laparoscopic subtotal cholecystectomy; NA, not available; OC, open cholecystectomy from the beginning; PTGBD, percutaneous transhepatic gallbladder drainage; SILS, single‐port laparoscopic surgery; WBC, white blood cell.

## 4 DISCUSSION

In this study we proposed a new SS to predict the necessity of the bailout procedure during LC after PTGBD, by using significant predictors of time until PTGBD after symptom onset and the maximal GB wall thickness. The SS for the prediction of the bailout surgery during LC was significantly associated with a risk of BDI. Despite no significant difference between patients with a score of 2 who underwent LC with the bailout procedure and OC, no patient who underwent the OC had BDI. The use of our novel SS to predict the necessity of the bailout procedure during LC after PTGBD was validated in a separate cohort. By comparing patients with a score of 2 with those with a score of 1 or less, the sensitivity and specificity of our SS for the prediction of the bailout procedure were 75.0% and 98.1%, respectively, and showed acceptable values.

Many previous studies have used factors, such as the open conversion rate, operating time, and incidence of complications as indicators of surgical difficulty.^4^ Focusing on the open conversion as an indicator of surgical difficulty, a meta‐analysis identified GB wall thickening (>4 to 5 mm) on ultrasound, male sex, advanced age, and obesity as risk factors for open conversion.^9^ In this study the maximal GB wall thickness was also found to be an independent predictor of the bailout procedure as open conversion, as in previous reports.^9^, ^10^ A thickened GB wall was previously reported to be predictive of a difficult exposure of the anatomy during LC because of increased inflammation, edema, and adhesions.^11^, ^12^ Tissue planes are less distinct and bleeding is more common due to repetitive inflammation episodes.^13^ Therefore, a thickened GB wall might present a more challenging laparoscopic dissection.

On the other hand, although it has been reported that a duration of symptoms of AC >72 hours was an independent predictor of open conversion as a bailout procedure, the time until PTGBD after symptom onset has never been reported.^14^ Koo et al reported that it would be easy and safe to dissect the tissue when performing LC within 72 hours of the onset of symptoms of AC because of the phase of edematous cholecystitis.^15^, ^16^ Rapid decompression of GB by drainage suppresses progression of inflammation.^17^ PTGBD in the phase of edematous cholecystitis seems effective because the inflammation of this phase is reversible.^18^, ^19^ Therefore, PTGBD within 3 days of the onset of symptoms might facilitate successful LC after PTGBD.

In this study, although there was no significant difference between patients with a score of 2 who underwent LC with the bailout procedure and those who underwent OC, none of the patients who underwent OC had BDI. In addition, among patients with a score of 2 who underwent LC with the bailout procedure, only patients who underwent open conversion had BDI, while patients who underwent LSC had no BDI. In TG18, LSC is an important procedure that should be considered in order to avoid serious damage to the BDI or blood vessels.^4^ However, LSC would require the skill of surgeons and might pose some problems such as postoperative bile leakage, recurrence of GB stone in the remnant GB, or a risk of concomitant GB cancer.^20^, ^21^ On the other hand, in a questionnaire survey of experts in Japan, South Korea, and Taiwan, only 17.5% of the respondents said that open conversion made surgery easier^22^ and open conversion might not necessarily be safe.^23^ The estimated incidence of serious complications, such as BDI or VBI, was reported to be two to five times higher for LC than for open cholecystectomy.^24^ In the current study with chronic cholecystitis after PTGBD for AC, two out of four patients with BDI had the accidents after the open conversion. The main reasons for BDI or VBI was misidentification of the anatomy caused by disorientation because of the adhesion from severe inflammation and intraoperative bleeding. Consequently, it might be better to select flexibly LSC or OC according to the skill of the surgeons for patients with a score of 2 after PTGBD because of the higher risks of BDI and needing open conversion.

The current study had several limitations. First, this was a retrospective study. Selection bias for the bailout procedure could arise because the decision to choose the bailout procedure might have varied greatly among surgeons, and the decision depended on the surgeons’ skill level. Second, most of the open cholecystectomies or LCs were performed according to the Tokyo Guideline 2013, in which conversion to open cholecystectomy was the only recommendation in patients with AC for which LC was difficult.^25^ No patient underwent LSC in the training cohort. According to a systematic review and a meta‐analysis, although postoperative bile leakage was more common following LSC than after open conversion, the rates of BDI, postoperative complications, and mortality were all lower.^20^, ^21^ In TG18, LSC with fenestrating or reconstituting and the fundus first technique should be chosen to prevent BDI according to the intraoperative findings.^4^ In the future, in patients with cholecystitis after PTGBD with a score of 2, outcome of the LSC should be prospectively compared with those of OC.

## 5 CONCLUSIONS

We proposed a new SS using the significant factors of time until PTGBD after symptom onset and the maximal GB wall thickness to predict the necessity of the bailout procedure during LC after PTGBD. Our SS for predicting the necessity of a bailout procedure correctly predicted the need and might be associated with a risk of BDI. Therefore, it might be better to select the flexibility of LSC or OC for patients with a score of 2 after PTGBD.

## DISCLOSURE

Funding: The authors received no funding support for this article.

Conflict of interest: The authors declare no conflicts of interest for this article.

Approval of the research protocol: The protocol for this retrospective research has been approved by a suitably constituted Ethics Committee of the institution and it conforms to the provisions of the Declaration of Helsinki. The Ethics Committee of The Jikei University School of Medicine (Tokyo, Japan), Approval No. 27‐177 (8062).

Informed Consent: Informed consent was not applicable.

Registry and the Registration number of the study: Registry of this study was not applicable.

Animal studies: Animal study was not applicable.
